# Gene and metabolite time-course response to cigarette smoking in mouse lung and plasma

**DOI:** 10.1371/journal.pone.0178281

**Published:** 2017-06-02

**Authors:** Mikaela A. Miller, Thomas Danhorn, Charmion I. Cruickshank-Quinn, Sonia M. Leach, Sean Jacobson, Matthew J. Strand, Nichole A. Reisdorph, Russell P. Bowler, Irina Petrache, Katerina Kechris

**Affiliations:** 1 Department of Biostatistics & Informatics, Colorado School of Public Health, University of Colorado Anschutz Medical Campus, Aurora, Colorado, United States of America; 2 Center for Genes, Environment, and Health, National Jewish Health, Denver, Colorado, United States of America; 3 Department of Pharmaceutical Sciences, School of Pharmacy, University of Colorado Anschutz Medical Campus, Aurora, Colorado, United States of America; 4 Division of Pulmonary, Critical Care and Sleep Medicine, National Jewish Health and University of Colorado, Denver, Colorado, United States of America; 5 Division of Biostatistics and Bioinformatics, National Jewish Health, Denver, Colorado, United States of America; 6 Department of Medicine, Indiana University, Indianapolis, Indiana, United States of America; Institute of Lung Biology and Disease (iLBD), Helmholtz Zentrum München, GERMANY

## Abstract

Prolonged cigarette smoking (CS) causes chronic obstructive pulmonary disease (COPD), a prevalent serious condition that may persist or progress after smoking cessation. To provide insight into how CS triggers COPD, we investigated temporal patterns of lung transcriptome expression and systemic metabolome changes induced by chronic CS exposure and smoking cessation. Whole lung RNA-seq data was analyzed at transcript and exon levels from C57Bl/6 mice exposed to CS for 1- or 7 days, for 3-, 6-, or 9 months, or for 6 months followed by 3 months of cessation using age-matched littermate controls. We identified previously unreported dysregulation of pyrimidine metabolism and phosphatidylinositol signaling pathways and confirmed alterations in glutathione metabolism and circadian gene pathways. Almost all dysregulated pathways demonstrated reversibility upon smoking cessation, except the lysosome pathway. Chronic CS exposure was significantly linked with alterations in pathways encoding for energy, phagocytosis, and DNA repair and triggered differential expression of genes or exons previously unreported to associate with CS or COPD, including *Lox*, involved in matrix remodeling, *Gp2*, linked to goblet cells, and *Slc22a12* and *Agpat3*, involved in purine and glycerolipid metabolism, respectively. CS-induced lung metabolic pathways changes were validated using metabolomic profiles of matched plasma samples, indicating that dynamic metabolic gene regulation caused by CS is reflected in the plasma metabolome. Using advanced technologies, our study uncovered novel pathways and genes altered by chronic CS exposure, including those involved in pyrimidine metabolism, phosphatidylinositol signaling and lysosome function, highlighting their potential importance in the pathogenesis or diagnosis of CS-associated conditions.

## Introduction

Chronic obstructive pulmonary disease (COPD) is a common, heterogeneous group of lung diseases characterized by airflow obstruction due to chronic bronchitis, emphysema, or small airways disease [1]. Cigarette smoke (CS) exposure is the primary risk factor for development of COPD, but COPD can persist and even progress despite smoking cessation. The ongoing and persistent alterations in gene expression induced by chronic CS exposure that may be linked to lung function decline and other manifestations of COPD remain to be elucidated.

In CS-susceptible mice strains emphysema-like enlargement of alveolar spaces and lung dysfunction that recapitulate mild human disease occur after six months of CS exposure [2]. To date, studies using diverse methodologies that assess changes in mouse lungs following CS exposure have identified mechanisms driving the development of COPD in smokers, including airway inflammation, oxidative stress, an imbalance of proteinases/anti-proteases, autoimmune responses, defective autophagy and injurious apoptosis of alveolar cells, ineffective clearance of apoptotic cells by macrophages, disrupted histone acetylation/deacetylation, and accelerated lung aging leading to failure of lung maintenance and repair [1,3].

Previous gene expression studies of CS-exposed mice have only moderate reproducibility, suggesting an influential role of various exposure protocols, technologies, analytical methods, and mouse strains. These gene expression studies have primarily utilized microarray technology, which has limitations compared to next-generation sequencing techniques due to sparser sampling of probes along the genome and reduced sensitivity to low expression levels of genes [4]. The experimental design, differential gene expression, and gene set enrichment of previous relevant gene expression studies are summarized in the Online Supplement (Tables A-C in S2 File).

Using a mouse model of prolonged CS exposure sufficient to cause airspace enlargement and increased lung compliance indicative of emphysematous COPD [5], we profiled the time course of lung transcriptome responses to CS in mice at varying time points up to 9 months of exposure with RNA-seq, including a smoking cessation group and age-matched controls. As an alternative to typical gene expression validation by RT-PCR or quantification of congruent changes of select gene products, in this study, we interrogated the functional relevance and potential for translation into biomarkers of lung transcriptomic changes related to metabolism by examining untargeted metabolomic profiles of matched plasma samples. Preliminary results from our study have been previously reported in the form of an abstract [6].

## Materials and methods

### Animals and exposure

All animal experiments were approved by the Institutional Animal Care and Use Committee at Indiana University, and all efforts were made to minimize suffering. Rodent suffering was monitored daily with use of a Body Condition scoring system. Three-month old female C57Bl/6 mice (Jackson Laboratories, Bar Harbor, ME, USA) were exposed using a whole body exposure system to either CS or ambient air control (AC) for 1 day, 7 days, 1 month, 3 months, 6 months, or 9 months as previously described (n = 5/group) [7]. A third group was exposed to 6 months of CS followed by a 3-month cessation period (SS group, for stop smoking); see experimental design in 1. At the end of experiments, the mice were euthanized by bilateral pneumothorax and exsanguination under 2–5% isoflurane together with 62.5 mg/kg ketamine and 9mg/kg xylazine. Plasma was collected by cardiac puncture and then snap-frozen in liquid nitrogen and stored at −80°C. Lungs were harvested as previously detailed [7]. Lung tissue was snap-frozen for RNA extraction and stored at -80°C. Assessment of lung function was performed on anesthetized mice using the Scireq flexiVent^™^ apparatus (Montréal, QC, Canada) and analyzed with Student’s t-tests (*p<*0.05) [5].

### RNA-seq library preparation and sequencing

Total RNA (500ng) was used to construct sequencing libraries using Illumina TruSeq Stranded mRNA LT Library Construction Kit, Catalog # RS-122-2101 (San Diego, CA, USA). Single-read 100 bp sequencing was performed on an Illumina HiSEQ 2000 instrument.

### Bioinformatics analysis

Sequence quality was assessed with FastQC (version 0.10.1). Sequences were mapped to the reference genome using GSNAP (version 2013-11-27) [8,9]. Uniquely mapped reads were quantified with HTSeq (version 0.5.4p3) [10]. Data are deposited in NCBI’s Gene Expression Omnibus (accession number GSE76205).

### Statistical analysis

Unless otherwise stated, a 10% false discovery rate (FDR) was used, and statistical significance was determined as *q≤*0.10 [11]. Analyses were performed in R (version 3.2.2) [12]. An independent filtering method was used across all time points [13]. See Online Supplement for details on all methods.

#### Individual time point analysis

The edgeR (version 3.10.5) exact test identified differentially expressed genes between AC- and CS-exposed groups at each time point. Significant results were filtered (fold change |FC|≥1.5).

#### Functional annotation and pathway analysis

Significantly enriched KEGG pathways were identified at each time point between AC- and CS-exposed mice with GAGE (version 2.18.0) [14].

#### Effect of cessation

The edgeR exact method identified modulated genes whose post-CS-exposure expression either reversed following cessation or persisted despite the cessation period (S1 Fig). Recovery genes, whose differential expression was unique to the SS versus CS comparison, were identified. Pathway analysis was performed as above for all pairwise comparisons.

#### Time series analysis

Linear models were fit for gene expression variables using time and group as categorical predictors. Using a protected testing procedure, orthonormalized polynomial contrasts were utilized to test linear and quadratic trends for time, as well as polynomial interactions between groups [15].

#### Differential exon usage

Reads were assigned to exon bins using HTSeq (version 0.6.0) and scripts provided by DEXSeq, and analyzed with DEXSeq (version 1.20.0) [16]. Time points were tested individually and filtered (|FC| ≥1.5).

#### Metabolomics analysis

Statistically significant pathways recurring at more than one time point from the RNA-seq results were probed to determine whether levels of metabolic compounds in these pathways were significantly different in the corresponding metabolome samples (*p*<0.05). Metabolite preparation and extraction methods have been described previously [7].

## Results

### Physiologic assessment of CS exposure on lung function

**Fig 1:**
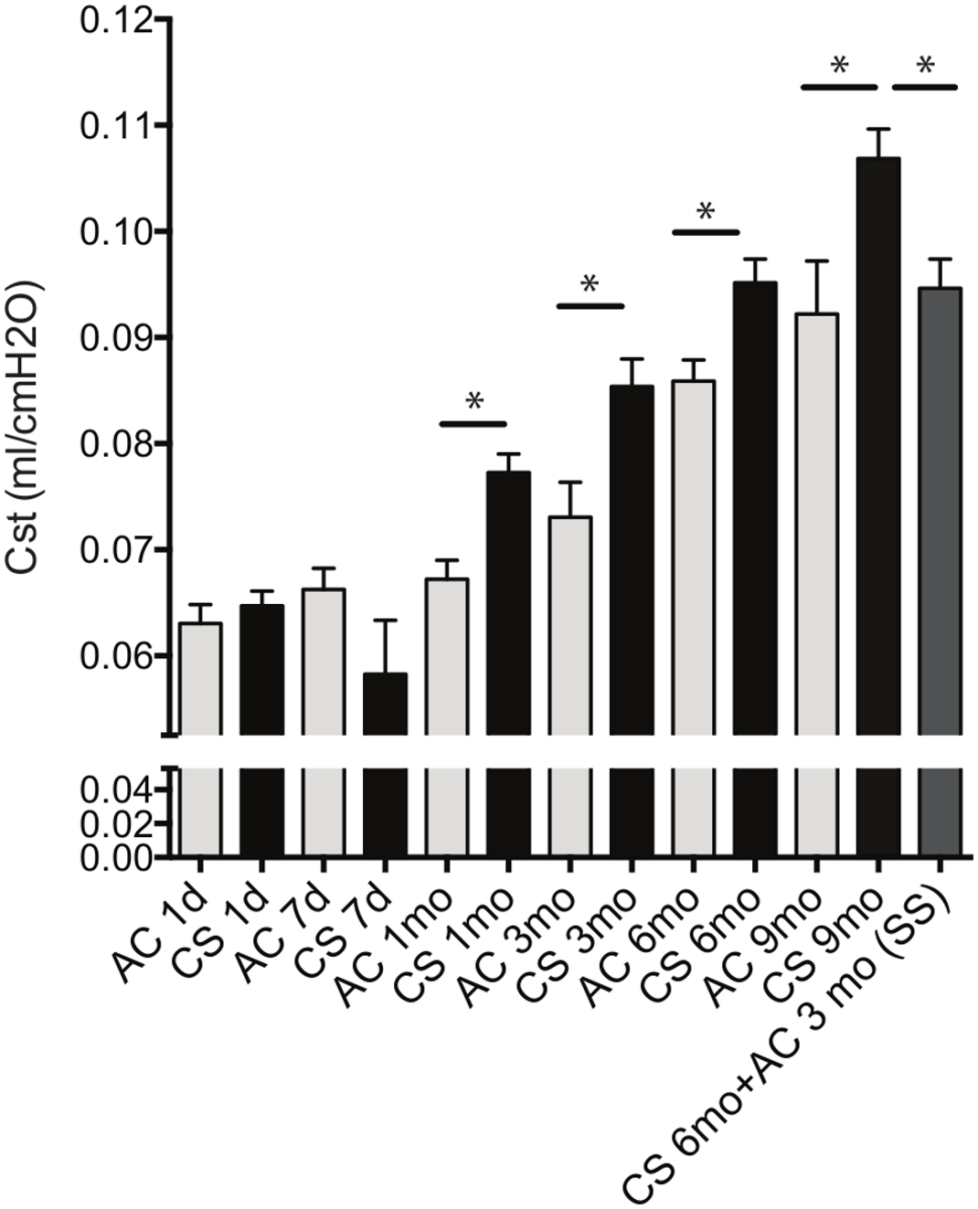
Mean static lung compliance (Cst). Cst measured by the flexiVent^™^ instrument for each experimental group at each time point. C57Bl/6 mice were exposed to ambient air (AC, grey bars) or chronic cigarette smoke (CS, black bars) for the indicated duration of time measured in days (d) or months (mo). In one group of mice that underwent smoking cessation (SS; stop smoking, dark grey bar), mice were allowed to recover at ambient air (AC) for 3 months following a 6 months CS exposure. N = 10 mice /group. Horizontal bars with asterisk indicate significant differences in mean static lung compliance (*p*<0.05). Bars represent the standard error of the mean.

To ensure that our CS exposure model induced expected emphysema-like lung injury in mice, we measured lung function of mice using the flexiVent^™^ apparatus. Chronic CS exposure for 3–6- and 9- months significantly increased static lung compliance compared to the AC littermates of similar age (Fig 1). These functional changes are consistent with a loss of lung elastance that characterizes emphysema-like lung injury in CS-susceptible strains of mice [5]. Since each CS-exposure group was age-matched for a control AC-exposed group, we had the opportunity to note that aging had a significant effect on lung compliance in these C57Bl/6 female mice (Fig 1). In a group of mice that was exposed to CS for six months, we stopped CS exposure for 3 months followed by assessment of lung compliance. At 3 months following CS cessation, lung compliance did not return to normal and although it remained higher (by ~ 5%) than respective age-matched unexposed littermates, the difference was not statistically significant, suggesting a more pronounced effect of aging rather than that of persistence of the CS exposure effect at this advanced age of the studied animals (12 months of age). Evaluation of lung histology was consistent with lung functional measurements and indicated that prolonged exposure to CS leads to progressive emphysema-like enlargement of airspaces compared to age-matched air controls (S2 Fig).

### Assessment of cigarette exposure

As additional confirmation of chronic exposure to CS, we examined the presence of nicotine derivatives in biofluids. Since nicotine itself has a short half-life, we examined hydroxycotinine and 4-oxo-4-(3-pyridyl)-butanoic acid in the plasma and broncho-alveolar lavage fluid (BALF; only available for day 1 and month 9 groups) respectively of the same mice that had their lung transcriptome profiled. We were unable to detect any nicotine metabolites in either the plasma or the BALF of any AC mice (S3 Fig). In contrast, with the exception of day 1, CS-exposed mice had evidence of nicotine derivatives in the vast majority of samples examined (S3 Fig).

### Differential gene expression induced by CS analyzed at individual time points

Of all 65 lung samples each representing distinct mice, three samples did not pass quality control (see Online Supplement for details) and were excluded from all subsequent analyses. Of the remaining 62 lung samples analyzed as a group, those collected at 1 month following AC- or CS exposures had lower overall transcript counts, more mapping ambiguity (due to reads mapping to multiple locations in the genome), and more rRNA when compared to the other time points. These observations were of sufficient concern to exclude the samples from intra-group comparison and subsequent time course analyses.

**Fig 2:**
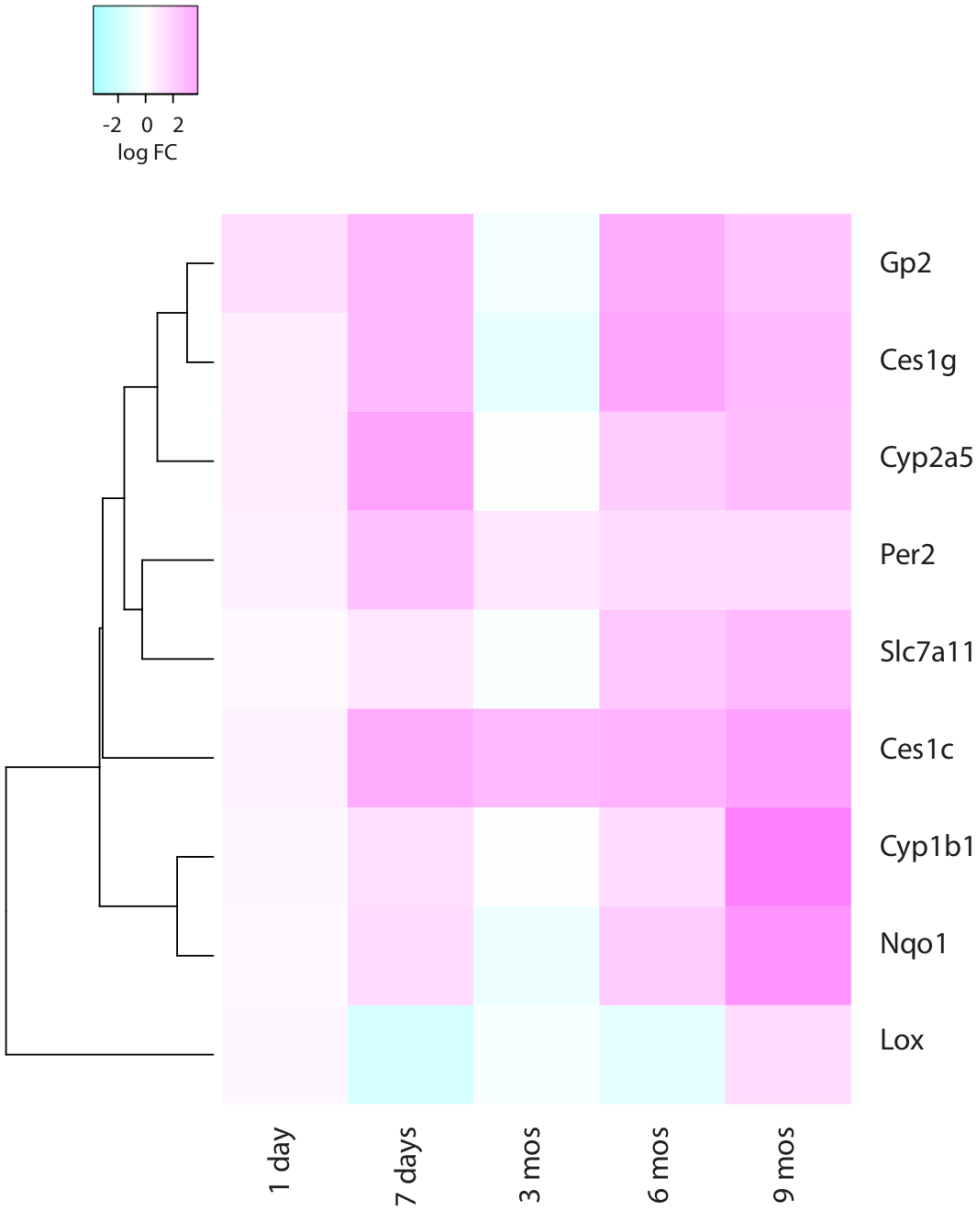
Heatmap of the relative change in gene expression. Log2 FC in CS-exposed mice compared to AC mice of the 9 genes found to statistically significantly differentially expressed at four of five time points (FDR≤0.10). Positive fold change (pink) indicates upregulation in CS-exposed mice; negative fold change (blue) indicates downregulation in CS-exposed mice.

CS exposure had the largest impact on total differential gene expression during the early and the late time points of CS exposure (Table 1, Tables D-J in S2 File). Furthermore, the differential expression of most genes did not persist beyond a single time point and no gene was identified as differentially expressed for more than four (of the five) time points. We noted only 9 genes that remained significantly differentially expressed (FDR≤0.10) with a |FC|≥1.5 for four time points, which we called persistently altered genes. The differential expression of these genes required more than 1 day of CS exposure (Fig 2). The persistently upregulated genes regulate xenobiotics metabolism, metabolism of cofactors/vitamins, lipid metabolism and circadian rhythm. Only one gene (*Lox*, which encodes for lysyl oxidase involved in crosslinking of collagens and elastin) was bi-directionally regulated, although upregulation was only observed at 6 months of CS exposure with most other time points showing significant downregulation (at 7 days, 3 months, and 9 months of CS exposure). Many of these genes in mouse that changed due to smoking exposure also showed expression differences in the lungs of emphysemateous subjects compared to controls (Table K in S2 File).

**Table 1:** Number of significantly differentially expressed genes, perturbed pathways and differentially used exons identified between CS- and AC-exposed mice, and between pairwise comparisons with SS (cessation group, for stop smoking) mice (FDR≤0.10, |FC| ≥ 1.5).

	*Time Point*					*Cessation*		
	1 day	7 days	3 months	6 months	9 months		CS vs. SS	AC vs. SS
***Genes***								
Up (CS)	6	733	132	85	376	Up (SS)	664	1
Down (CS)	4	769	225	26	486	Down (SS)	705	2
***Enriched Pathways***								
Up (CS)	55	12	8	3	20	Up (SS)	68	17
Down (CS)	10	71	0	0	35	Down (SS)	25	5
***Exons***								
Up (CS)	54	36	7	49	2	Up (SS)	43	26
Down (CS)	21	13	7	5	17	Down (SS)	241	480

### Pathways analysis of differential gene expression induced by CS

The analysis of differential gene expression by gene set enrichment using KEGG revealed several cellular pathways that were significantly perturbed by CS exposure at each time point (Table 1). However, the majority (38 of 55) of pathways enriched for upregulated genes after 1 day of CS exposure were then significantly enriched for downregulated genes after 7 days of CS exposure (S4 Fig) and included genes known to be involved in cancer, immunity, signal transduction, cellular community, infectious diseases and digestive system processes, with those involved in immunity remaining enriched for downregulated genes again after 9 months of CS-exposure. Of the 10 pathways enriched for downregulated genes by brief CS exposure (1 day), half displayed enrichment of upregulated genes after 7 days of CS exposure and again after 9 months. These pathways represent biological processes involved in protein translation, energy metabolism, and neurodegenerative disorders (S4 Fig).

**Fig 3:**
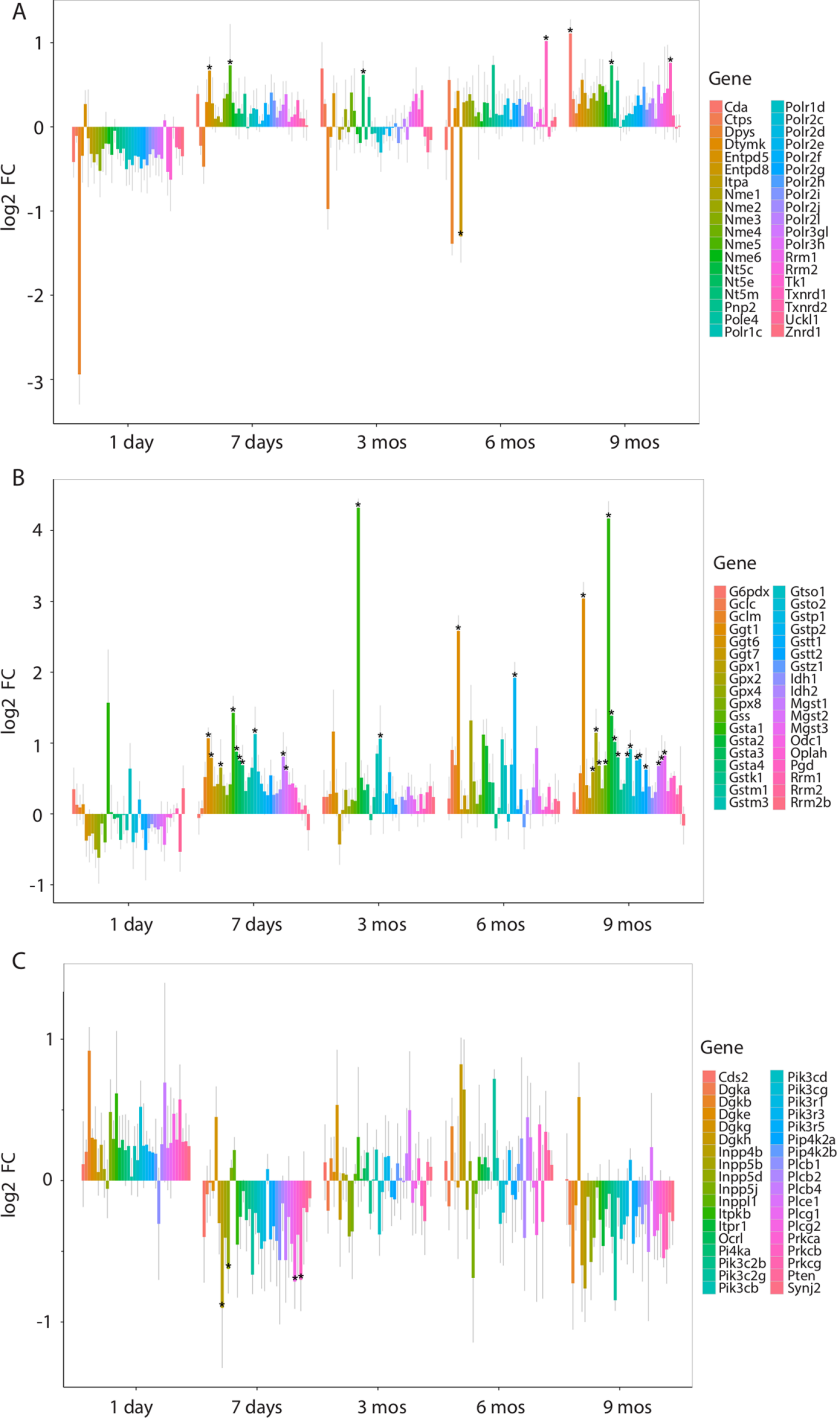
Behavior of influential genes in relevant metabolic pathways. Expression was summarized as base-2 fold change (log2 FC). Influential genes determined by GAGE using coreGeneSets, and are those member genes that substantially contribute to the significance of the entire pathway [14]. (A) Pyrimidine metabolism was initially enriched for downregulated genes, but later enriched for upregulated genes at 9 months of CS exposure. A gradual transition from downregulation to upregulation is apparent, especially for the gene *Dpys*. (B) Glutathione metabolism was the most consistently enriched KEGG pathway for upregulated genes. While 1 day of CS exposure did not elicit upregulation in many genes, at 7 days of CS most of the influential genes were upregulated. (C) Phosphatidylinositol (PI) signaling system was initially enriched for upregulated genes, but later enriched for downregulated genes at 9 months of CS exposure. Grey bars indicated 95% confidence intervals.

3 displays the relative change in base-2 normalized expression values (log2 FC) for core genes in three of the recurring significant pathways identified at multiple time points. The pyrimidine metabolism pathway, a nucleotide metabolism pathway, was initially enriched for downregulated genes, later to be enriched for upregulated genes after 9 months of CS exposure (Fig 3A). The pathway most consistently enriched for upregulated genes throughout CS exposure was that of glutathione metabolism (Fig 3B). The phosphatidylinositol (PI) signaling system pathway was initially enriched for upregulated genes after 1 day of exposure, later to be enriched for downregulated genes after 9 months of CS exposure (Fig 3C).

**Fig 4:**
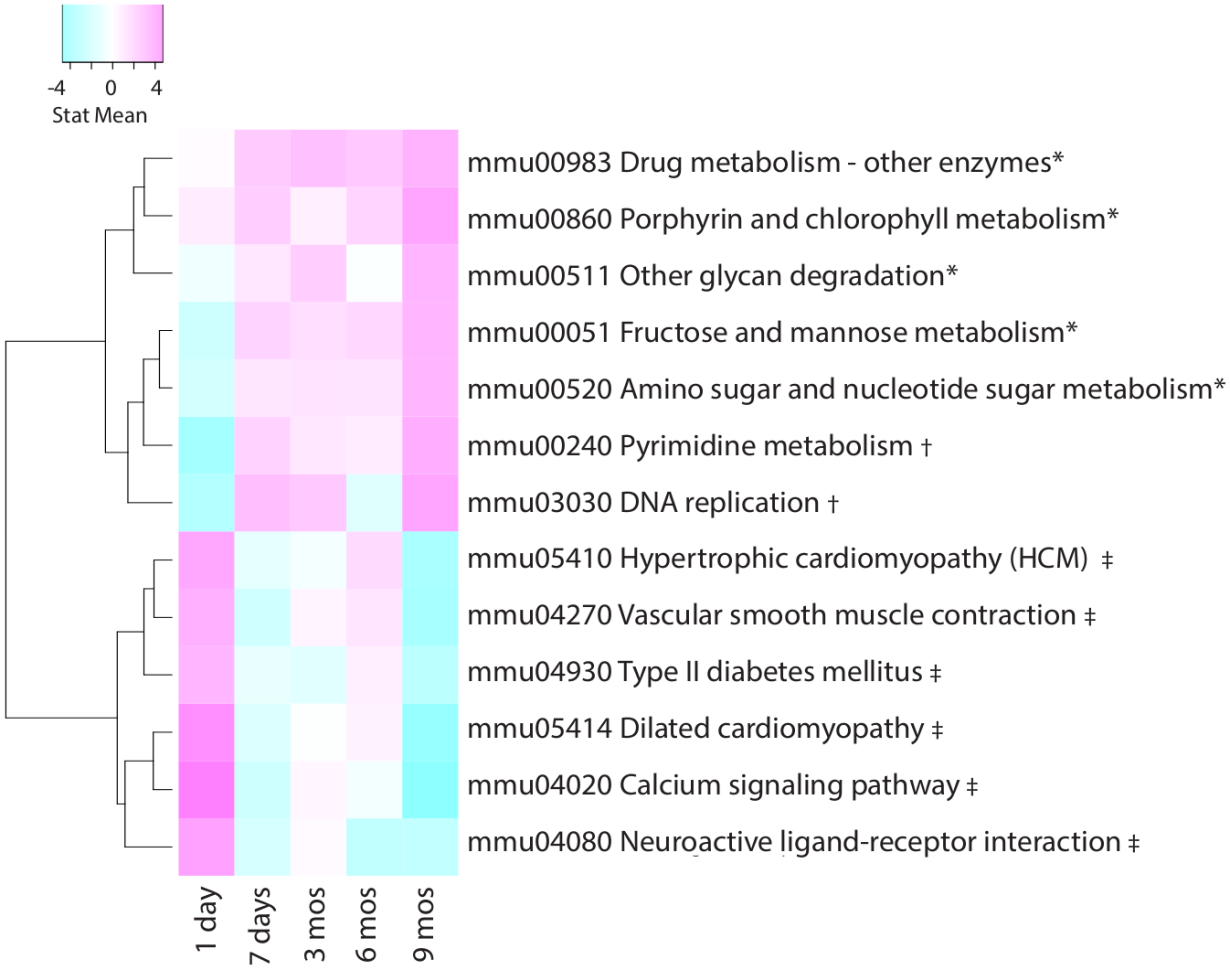
KEGG pathways displaying unique enrichment patterns at 9 months of CS (FDR≤ 0.10). The pathways were either exclusively differentially enriched or exhibited an opposite direction of enrichment after 9 months CS exposure compared to other time points. *Denotes pathways enriched for upregulated genes after 9 months of CS exposure, but was not significantly enriched at any other time point. †Denotes pathways enriched for upregulated genes after 9 months of CS exposure, but initially enriched for downregulated genes after 7 days; ‡ denotes pathways enriched for downregulated genes after 9 months of CS exposure, but initially enriched for upregulated genes after 7 days of CS exposure. No pathways were significantly enriched for downregulated genes after 9 months that were not significantly enriched at any other time point.

Since few studies investigated transcriptional changes beyond six months of CS exposure, we analyzed pathways that were uniquely enriched following 9 months of CS exposure and identified five pathways enriched for upregulated genes involved in the metabolism of carbohydrate, glycan, cofactors and vitamins, and that of xenobiotics (4). The pathways that changed directionality of enrichment at this time point compared to acute (1day) CS exposure were enriched for significantly downregulated genes involved in signal transduction, cardiovascular, endocrine and metabolic disorders and were enriched for upregulated genes involved in pyrimidine metabolism and DNA replication pathways (Fig 4).

### Alterations in the lung transcriptome after smoking cessation

Of the 882 genes that were differentially expressed between AC and CS mice after 9 months of exposure, 99.7% were fully or partially reversible at 3 months following removal from CS exposure (Table 2, Tables H-J in S2 File). Only one gene, *Rasd1*, that encodes for a small GTPase was identified as having a fully persistent pattern, remaining differentially downregulated compared to age matched AC-exposed control mice, while *Ggt1*, involved in glutathione metabolism, remained partially upregulated, and *Pdk4*, involved in the glucose metabolism, remained partially downregulated (S1 Fig for cessation patterns).

**Table 2:** Abundance of genes found significantly differentially expressed using edgeR (FDR≤0.10) between CS- and AC-exposed mice and enriched pathways using GAGE (FDR≤0.10) exhibiting patterns detailed in S1 Fig. Normalization, filtering, and modeling was performed using data from three groups (AC, CS, and smoking cessation).

	Reversible	Semi-reversible	Persistent	Semi-persistent	Continuing
***Genes***					
**Up (CS)**	278	134	0	1	0
**Down (CS)**	269	198	1	1	0
**% of Total**	62.00%	37.64%	0.11%	0.23%	0.00%
***Enriched Pathways***					
**Up (CS)**	18	5	0	1	0
**Down (CS)**	38	1	0	0	0
**% of Total**	88.89%	9.52%	0.00%	1.59%	0.00%

Pathway analysis similarly revealed that 98.4% of the pathways significantly perturbed between AC and CS- exposed mice were fully or partially reversible, primarily related to immune system processes, cardiovascular disease, signal transduction, and metabolism. The only semi-persistent pathway was the lysosome pathway, enriched for upregulated genes encoding lysosomal components.

We identified 820 genes that were uniquely expressed following smoking cessation, which may encode responses in gene expression that are only triggered after prolonged exposure followed by cessation, termed “recovery” responses by a previous investigation of transcriptomic changes during smoking cessation [17]. The “recovery” pathways enriched for upregulated genes are involved in infectious diseases, cancers, innate immunity and endocytosis; those enriched for downregulated genes are involved in amino acid, lipid, and carbohydrate metabolism (Table L in S2 File). In addition, many of the genes showing differences following cessation compared to the mice still exposed to smoking (Table I in S2 File) also show expression differences in lungs of former smokers compared to current smokers (Table M in S2 File).

### Time trend analysis of differential gene expression induced by CS

We next analyzed our results across time to identify genes with group-by-time interactions, where the changes in gene expression profiles across time were significantly different and dependent on the treatment group. For the remaining 5 time points after dropping data from 1 month, 192 genes showed significant polynomial trends for time or group-by-time interactions (Table N in S2 File). There were no genes displaying only flat expression trajectories (no effect of time) while having a significant effect of treatment group across all time points, indicating that differences between AC and CS mice are variable over the time. Fourteen genes displayed a significant time effect that did not differ between treatment groups, even though there was a significant group effect. Eleven of those 14 genes, including *Grm4* and *Fam162b*, displayed significant group effects plus linear time effects, but no interaction, resulting in parallel linear trends. *Grm4*, which encodes for the metabotropic glutamate receptor 4, exhibited a significant positive trend for time, with the CS-exposed mice showing generally lower expression levels than AC-exposed mice, possibly indicating an aging effect. *Fam162b*, which encodes for a yet to be characterized transmembrane protein, exhibited a negative trend for time, with CS-exposed mice displaying higher expression levels than AC-exposed mice. Three of those 14 genes with significant group plus time effects displayed significant group effects plus quadratic time effects, where gene expression levels showed vertically translated u-shaped trends with time, rather than strict linear trends.

**Fig 5:**
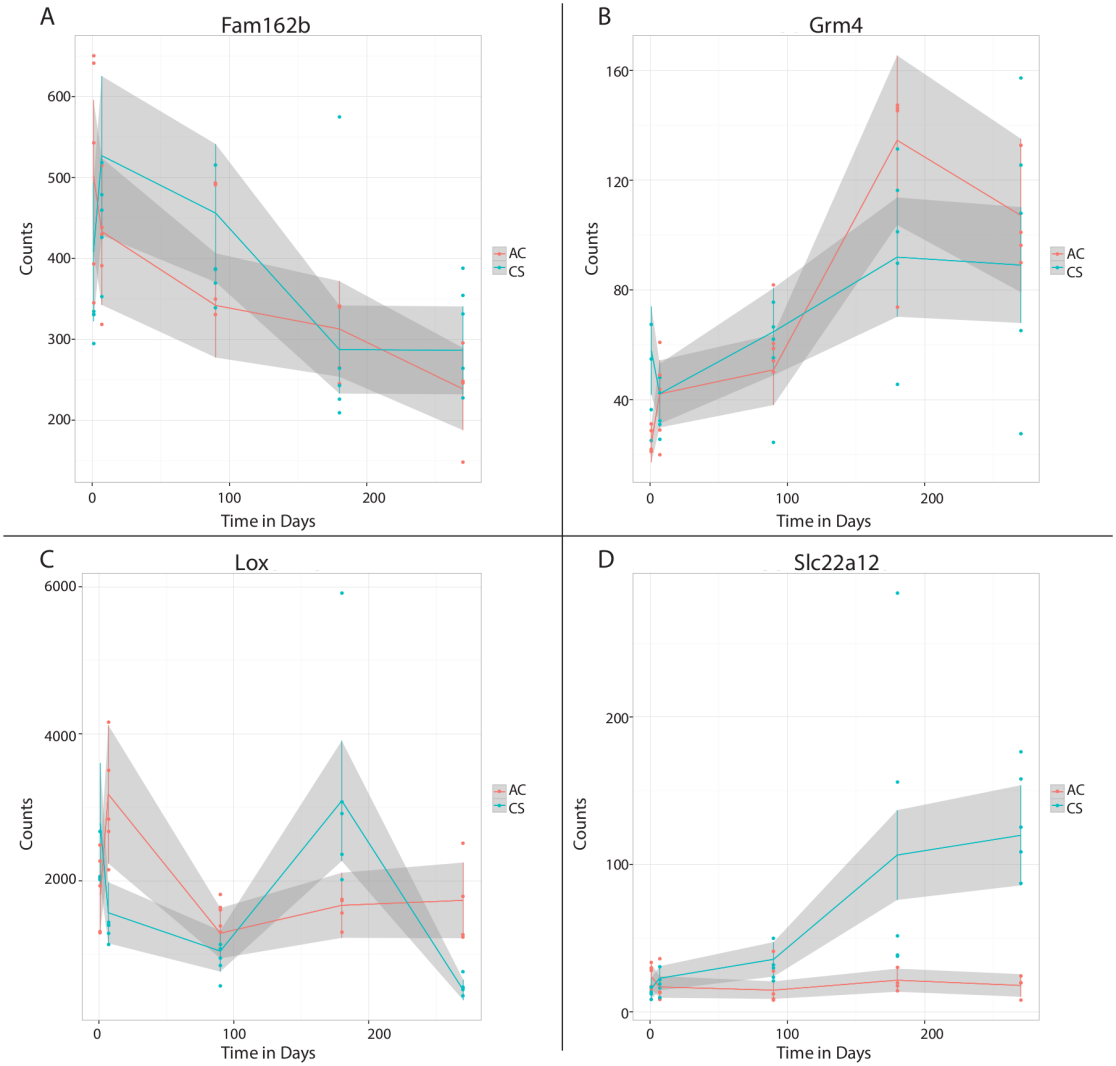
Genes showing a previously unsuspected significant time trend with CS exposure. (A) *Fam162b* shows a significant downward trend for linear time and significant effect of group. (B) *Grm4* shows significant upward trend for linear time and significant effect of group. (C) *Lox* was bi-directionally regulated in individual time point analysis. (D) *Slc22a12* displayed significant linear time by treatment interaction, with no change in expression in the AC group.

Another set of genes showed group dependent effects of time, having statistically significant group-by-time interaction terms. These interactions indicate genes where the effect of CS depends on the time point. The interactions reflect more complex gene expression profiles than those profiles that have a trend across times regardless of CS exposure. Fifteen genes, including *Ggt1* and *Slc22a12*, displayed significant linear time by treatment group interaction, both being relatively flat in AC-exposed mice and increasing through the 9 months of CS exposure. There were 78 genes, including *Lox*, displaying significant interactions between quadratic time and treatment group. Fig 5 displays time trends for selected genes.

### Differential exon usage induced by CS

We examined differential exon usage since genes that are regulated by this mechanism may functionally differ from genes that are regulated by differential gene expression. This analysis identified 219 differentially used exons across all time points associated with 226 gene symbols, 190 of which were not identified by the differential gene analysis (Table 1). Only two exons were identified at more than one time point to exhibit differential exon usage between CS- and AC-exposed mice. On the *Lipe* gene, which encodes for hormone-sensitive lipase, an exon (ENSMUSE00000824059/ENSMUSE00000665329) contained in transcript 003 (ENSMUST00000149349) or transcript 201 (ENSMUST00000105177) was significantly upregulated in CS-exposed mice at 7 days and 9 months of exposure. On the *Agpat3* gene, which encodes for an acyltransferase involved in phospholipid biosynthesis, exon ENSMUSE00000666029 was significantly downregulated in CS-exposed mice at 7 days and 9 months of exposure. This exon is unique to transcript 003 (ENSMUST00000105389), indicating a switch to one or more alternative transcripts (S5 Fig). Although in the whole gene analysis *Agpat3* was not found to be differentially expressed at any time point, *Lipe* was differentially upregulated after 7 days, 3 months, and 9 months of CS exposure. While no significantly enriched gene ontology (GO) molecular function categories [18,19] were found for the genes exhibiting differential exon usage for CS compared to AC, the differential exon usage resulting from recovery responses from smoking (Table 1) identified pathways significantly enriched for the GO molecular function categories of cytoskeletal protein binding, cation binding, motor activity, nucleoside-triphosphate regulator activity, GTPase regulator activity, actin binding, metal ion binding, and ion binding (FDR≤0.10).

### Changes in plasma metabolome associated with CS-induced transcriptome

As many of the top differentially expressed genes and pathways in response to CS exposure were related to energy metabolism and other metabolic pathways, we interrogated the association between lung transcriptomic and plasma metabolomic perturbations using untargeted metabolomics of paired plasma samples. Such association may reflect functionally active lung or systemic transcriptional responses induced by CS exposure. Six pathways (Fc gamma R-mediated phagocytosis, pathways in cancer, leishmaniasis, gap junction, PI signaling system, and pyrimidine metabolism) were identified as concordantly significantly altered at multiple time points in both the lung transcriptomic and untargeted plasma metabolomic data (Table 3, Table O in S2 File, S6 Fig). The metabolites belonging to pyrimidine metabolism and PI signaling pathways remained significantly altered even after 3 months of smoking cessation, despite reversal of transcriptomic signals. Conversely, the potent lung transcriptomic changes in glutathione metabolism pathway did not translate in significant corresponding metabolomic changes in plasma, although several metabolites in that pathway were individually significant (S7 Fig).

**Table 3:** Pathways overlapping between transcriptomic and metabolomic data. Checkmarks signify significant pathway perturbation in metabolomic data (*p*<0.05), pink shading denotes enrichment for upregulated genes in that pathway in CS-exposed mice; blue shading indicates enrichment for downregulation in CS-exposed mice (FDR≤0.10). Perturbations indicated by shaded fields under the “Cessation” column persisted following cessation when compared to AC mice.

	1 day	7 days	3 months	6 months	9 months	Cessation
Oxidative phosphorylation				✓		
Parkinson's disease						✓
Pyrimidine metabolism					✓	✓
Leishmaniasis		✓	✓	✓		✓
Fc gamma R-mediated phagocytosis	✓	✓	✓	✓		✓
Inositol phosphate metabolism					✓	
Gap junction	✓	✓	✓			
Pathways in cancer	✓	✓	✓	✓		✓
Phosphatidylinositol (PI) signaling system	✓	✓	✓	✓	✓	✓

We discovered two pathways (pyrimidine metabolism and PI signaling system) not previously shown to be dysregulated in COPD, as differentially regulated by CS for both the lung transcriptome and plasma metabolome. In the pyrimidine metabolism pathway, deoxythymidine 5’-diphosphate (dTDP), glutamine, uridine, cytidine, cytosine, deoxycytidine, uracil, and urea metabolites were statistically significantly altered in CS-exposed mice (*p* <0.05). After 1 day of exposure, significantly higher levels of deoxycytidine were observed in CS-exposed mice. At 9 months CS exposure, we observed significantly lower levels of uridine and uracil in CS-exposed mice compared to controls that persisted despite cessation (Table O in S2 File). The PI signaling system pathway showed increased levels of diglycerides or diacylglycerols (DG) and phosphatidic acids (PA) after 1 day of CS exposure. DGs tended to remain significantly increased, but after 7 days and 9 months of exposure PIs significantly decreased in CS-exposed mice.

### Changes in BAL fluid metabolome associated with plasma metabolome and CS-induced transcriptome

We performed additional metabolomics profiling on BALF collected at day 1 and month 9 following CS exposure and respective control mice. Remarkably, all the enriched pathways for month 9 overlap with pathways identified in the lung transcriptome (Table P in S2 File). In addition, many also overlap with the pathways identified in the plasma metabolome. These results indicate that the BALF and plasma metabolomes may be informative for transcriptomic lung changes.

## Discussion

We report the first next-generation sequencing (NGS) gene expression study of a mouse model of sustained loss of lung elastance following chronic CS exposure. NGS RNA-seq is a more powerful, comprehensive, and sensitive technology than the microarrays used previously. In addition, our study is, to our knowledge, one of the first to integrate high-throughput RNA-seq data with high-throughput metabolomic data and the only one to do so utilizing paired lung-plasma samples in a time course fashion (Tables A-C in S2 File).

### Pathways identified by integrated genomic-metabolomic approach

One of the main novel findings is that CS exposure significantly modifies the expression of genes in the pyrimidine metabolism pathway and these changes reverberate in the plasma metabolome, as noted using paired metabolomics approach. The influence of CS exposure on pyrimidine metabolism has been reported using ^1^H-NMR metabolomics where mice acutely exposed to CS had elevated uridine, but other pyrimidine metabolite levels were decreased [20]. In our study, both uridine and uracil were significantly decreased in CS-exposed mice following 9 months of exposure and remained decreased despite smoking cessation. These two metabolites are integral components of pyrimidine degradation and salvaging, rather than *de novo* biosynthesis. Their diminished levels in CS-exposed mice may suggest uridine and uracil are being re-integrated to a greater extent during pyrimidine salvage [21]. Transcriptomic evidence supports increasing pyrimidine salvage activity with extended CS exposure, since significant upregulation of *Cda*, which encodes cytidine deaminase, a pyrimidine salvaging enzyme, was also noted after 9 months of CS exposure. It is conceivable, that because it is more energy efficient, the pyrimidine salvage pathway is preferentially engaged over that of *de novo* synthesis during chronic CS exposure, but further studies are needed to demonstrated this. However, the pyrimidine salvage pathway appears engaged only following chronic CS exposures, since acutely CS decreased the expression of the gene encoding for conversion of 5,6-dihydrouracil to 3-ureidopropionate, *Dpys*. This finding, along with the increased plasma deoxycytidine, a metabolite recycled by mitochondrial and cytosolic nucleoside kinases to limit the need for *de novo* synthesis of pyrimidine [21], indicated inefficient pyrimidine recycling and salvage during acute CS exposure. The signal for increased pyrimidine pathway activity during chronic CS exposure may reflect increased DNA replication and repair that persists following smoking cessation.

A second novel pathway identified dysregulated after CS exposure was the PI signaling system, where we observed bidirectional regulation (initial increase in expression, followed by a decrease) of several genes. These were paralleled by significantly decreases in PIs plasma levels at several sub-acute and chronic time points of CS exposure. The relevance of these changes to emphysema development may be related to increased cellular sensitivity to DNA damage, since studies in yeast suggest that defects in PI metabolism, through a yet to be determined mechanism, are associated with defects in DNA integrity checkpoints and sensitivity to DNA damage [22].

In addition to the identification of novel pathways, this study demonstrates that a paired NGS genomic-metabolomic approach can confirm some previously reported pathways. For instance, genes involved in glutathione metabolism, *Nfr2* client genes, ubiquitin proteasome, growth factors, extra cellular matrix, apoptosis, cell survival, and detoxification have been shown to be upregulated in acutely exposed mice previously (Table C in S2 File) [23,24]. Enrichment for upregulated genes relating to glutathione metabolism and xenobiotics metabolism was not apparent until 7 days of CS exposure in our study. The upregulation of the glutathione metabolism genes in the lung was, however, not translated into congruent changes in the respective metabolites in plasma, although previous reports do identify corresponding changes in glutathione-related metabolites in the bronchoalveolar lavage fluid following sub-chronic exposure to CS [25].

### Time-specific effects of CS exposure on gene regulation

When analyzed by individual gene, our results indicated that the acute response to CS (during the first 7 days of exposure) is extinguished and replaced with distinct differential gene responses as the CS exposure progresses to longer durations, spanning months. Interestingly, none of the pathways enriched for upregulated or downregulated genes at day 1 remained similarly regulated at day 7 of CS exposure. In turn, those pathways changed direction of gene enrichment after 7 days of CS exposure, a pattern recapitulated by the respective pathways detected in plasma using metabolomics. Since most of the previous studies of acute CS exposure investigated a single time point, typically after 1 day of CS exposure [23,24], or were limited to a select panel of genes [26], our study is the first to detect this shift in gene regulation that occurs within the first seven days of CS exposure.

Similar to previous reports [27,28], our pathways analysis showed that changes induced by sub-chronic CS exposure were dominated by upregulation of genes related to immune responses, oxidative stress responses, and xenobiotics metabolism. After 3 months of CS exposure the enrichment for upregulated genes in our study was related to drug metabolism, xenobiotics metabolism and glutathione metabolism. In our study, we did not observe any pathways enriched for downregulated genes at 3 months of CS exposure, although previous studies had identified downregulation of genes relating to transcription, signal transduction, and transport functions [27]. We did, however, observe persistent significant enrichment of the PI signaling system pathway in the metabolomic data during the subchronic stage of exposure, a pathway involved in signal transduction and environmental information processing (Table 3).

During chronic CS exposure, the pathways most significantly enriched for downregulated genes belonged to immune system processes and immune diseases, whereas pathways enriched for upregulated genes were mostly related to neurodegenerative disorders, transport, and catabolism. The persistent alteration of *Ces1g* and *Ces1c* and the *Cyp1b1* and *Cyp2a5* gene expression during the chronic stage was one the most significant results, indicating a shift in energy metabolism. In our study, Fc-gamma R-mediated phagocytosis, an immunity pathway that plays a role in the uptake and destruction of pathogens, was enriched for downregulated genes after 9 months of CS exposure, and the pathway for the neurodegenerative Parkinson’s disease was enriched for upregulated genes. Both of these pathways were reversible upon cessation. In other cases, the exon analyses identified alternative splice sites or differential exon usage not detected by differential gene expression analysis [29]. Using this methodology, we identified that an exon differentially expressed in *Agpat3* at 9 months is a 3’-UTR, which predicts that the coding sequence is unlikely to be affected, but the transcript switch may influence the mRNA localization, stability, export, and translation efficiency. *Agpat3* encodes for lysophosphatidic acid acyltransferases which catalyze the acylation of lysophosphatidic acid to phosphatidic acid, an important step involved in glycerolipid metabolism [30].

### Persistent effects of CS exposure on gene regulation

Despite the complexity of the changes induced in the lung following chronic CS exposure, multiple studies have identified only a few dozen individual genes that are differentially expressed (usually consistently upregulated) throughout acute, sub-chronic, and chronic CS exposure (Table B in S2 File). Our study agrees with these findings, identifying only 9 genes that are significantly and persistently altered. Several of these genes confirm previous microarray findings of consistent *Nqo1*, *Cyp1b1*, *Slc7a11*, and *Per2* upregulation during subchronic and chronic CS exposure [23,24,31,32]. Two of the persistently altered genes, *Lox* and *Gp2*, have not been previously implicated in mouse models of active chronic CS exposure. The persistent upregulation of *Gp2*, a gene expressed in goblet cells [33] may link it to the heightened mucosal immune response induced by CS exposure [17]. *Lox*, the only significantly bi-directionally persistently altered gene encodes the precursor protein lysyl oxidase, required to crosslink collagen and elastin, which makes it critical to vascular and extracellular matrix remodeling. Although in vitro experiments demonstrated that *Lox* expression decreases with exposure to tobacco-specific carcinogens [34], it is unclear what explains the bidirectional regulation and the role of lysyl oxidase in airway or vascular remodeling in COPD remains to be demonstrated.

We also identified several persistent changes using analyses for polynomial trends for time and group-by-time interactions, which allowed insights into gene expression temporal patterns where the effect of CS depends on the time point (Fig 5). This analysis identified a novel association of CS exposure with *Fam162b* expression, a gene predicted to encode an integral membrane protein [35]. *Slc22a12*, a urate transporter has not been previously associated with COPD. The parallel increase in *Slc22a12* expression with the duration of CS-exposure may indicate a progressive acceleration of the purine metabolism, given that urate is the end product of purine metabolism. Taken together with our observations concerning the pyrimidine metabolism pathway, nucleotide metabolism may play an integral role in the development of COPD.

### Smoking cessation reverses CS effects on gene regulation

Nearly all gene expression changes between AC- and CS-exposed mice were reversible following smoking cessation, similar with earlier reports using microarray technology [17,36,37]. The unique “recovery” gene pathways were enriched for downregulated genes involved in carbohydrate and amino acid metabolism pathways, and for upregulated genes related to innate immunity, infectious diseases, and cancers. Of these, previous studies have only identified genes primarily related to innate immunity and inflammatory response as being differentially regulated during this recovery phase [17]. Regarding individual gene expression during this recovery period, we noted significant downregulation of *Pdk4* and *Rasd1*, both identified as inactivated tumor suppressor genes in a global DNA methylation study [38]. The semi-persistent and persistent downregulation of *Pdk4* and *Rasd1*, respectively, may indicate ongoing cell proliferation required for repair after smoking cessation. Their role in tumorigenesis in the context of smoking and smoking cessation has not been investigated. Of the 63 pathways, only one, the lysosome pathway, failed to show reversibility of CS-induced changes.

## Conclusions

One limitation of our study may be related to the exclusion from analysis of the 1month CS exposure samples due to lower sequencing quality of the samples collected after 1 month of CS exposure compared with other time points. In addition, the unequal study treatment group sizes at 1 day, 6 months, and 9 months could have decreased the power to detect differential gene expression. Our results indicate several common responses including oxidative stress response and reversible changes after cessation. However, there are some other pathways, such as “leishmaniasis” (Table 3) related to parasitic disease, which has no direct connection with CS exposure [39]. After 7 days of exposure, the leishmaniasis pathway was dominated by downregulation of multiple DUSP and MAPK family genes as indicated by the coreGenes function in GAGE. The DUSP family of genes is a proposed modulator of MAPK signaling, which may in turn modulated adaptive immune responses [39]. Thus, the significance of this pathway may be due to common overlapping inflammatory and immune responses to different disorders or to biases in the pathway databases based on current, but not necessarily complete annotation. Although our study is one of the most comprehensive expression studies of CS exposure in mouse, the differential exon analysis was likely underpowered due to sample size and sequencing depth to identify differential exons, since ten times more exons are tested than genes. We also acknowledge that there is not always a direct link between transcripts and metabolites [40], especially when correlations are made between genes expressed in an inhomogeneous mix of multiple cell types represented in whole lung tissue and metabolites in plasma. However, at acute and chronic time points we did find overlap in perturbed pathways between the plasma metabolome, BALF metabolome and lung transcriptome. Finally, the results on the BALF metabolomics profiling may be reflecting increased macrophage numbers in mice exposed to smoke. While we do not have macrophage cell-type specific counts, total BALF and other cellular counts did not show marked changes in cell numbers and percentages between groups (data not shown).

Indeed, our analysis did not allow the identification of cell type-specific signals induced by CS exposure, but the presence of a significant signal despite the multi-cell origin and potential dilution of weaker cell-specific signals increases the relevance of our results. Our results can stimulate future cell-specific analyses as well as mechanistic investigation to understand the role of the identified pathways in the development of various aspects of COPD. Finally, one of our main findings is that the acute response at day 1 was significantly distinct and for some genes, even unique, compared to later time points of CS exposure. This is consistent with different stages of adaptation of the oxidant/antioxidant responses noted in the literature, where drastic changes in the lung with acute smoking become less apparent or have a reverse trend over time, such as with glutathione, which is depleted after acute smoking exposure and then increases with chronic smoking exposure [41].

In conclusion, our study demonstrates that profiling transcriptome changes at multiple time points with RNA-seq provides evidence that acute responses to CS are varied and transient, and select genes and pathways remain consistently modulated over time and persist despite smoking cessation. Using an integrated “omics” approach, we revealed pathways related to signal transduction and nucleotide metabolism that translate to plasma metabolic profiles and indicate active DNA damage and repair in response to CS, which may be linked to the development of COPD.

## Supporting information

S1 File Extended materials and methods. (DOCX)

S2 File **Table A**. Experimental design for relevant gene expression studies of CS exposure in mouse model. **Table B**. Differentially expressed genes from relevant gene expression studies of mouse model of CS exposure **Table C**. Significantly enriched gene sets and pathways from relevant gene expression studies of mouse model of CS exposure **Table D**. Differentially expressed genes: Day 1 **Table E**. Differentially expressed genes: Day 7 **Table F**. Differentially expressed genes: Month 3 **Table G**. Differentially expressed genes: Month 6 **Table H**. Differentially expressed genes: Month 9 CS vs AC **Table I**. Differentially expressed genes: Month 9 SS vs CS **Table J**. Differentially expressed genes: Month 9 AC vs SS **Table K**. Comparison with Human Lung Gene Expression in Emphysema Subjects **Table L**. Enriched KEGG recovery pathways unique to the CS vs SS comparison after 9 months of CS exposure (FDR≤0.10). **Table M**. Comparison with Human Lung Gene Expression in Former and Current Smokers **Table N**. Lists of genes displayed significant temporal trends over time by trend type **Table O**. Pathway analysis in plasma metabolome **Table P**. Pathway analysis in BALF metabolome.
(XLSX)

S1 Fig Patterns of gene regulation. Within each pattern, the white squares represent a pattern of upregulation in CS- vs. AC-exposed mice, and black squares represent a pattern of downregulation in CS- vs. AC-exposed mice. Comparisons with asterisks represent differences that must be statistically significant for the cessation pattern to hold. We defined as fully reversible genes those that were significantly differentially expressed between in CS-exposed mice compared to AC and that were differentially expressed in SS (stop smoking or smoking cessation) in an opposite direction compared to CS-exposed mice, but not differentially expressed in SS compared to AC. Compared to fully reversible patterns, semi-reversible genes did not fully return to AC levels, being differentially expressed between AC and SS mice. Fully persistent genes were significantly differentially expressed in CS vs. AC, and AC vs. SS, but not between SS and CS mice. Semi-persistent genes were similar to fully reversible genes, except they were differentially expressed between CS and SS mice. Continuing patterns were defined as higher differential expression in AC vs. SS comparison compared to the AC vs. CS comparison.
(TIFF)

S2 Fig Lung parenchyma histology of mice chronically exposed to cigarette smoke. Airspaces of mouse lungs imaged (10x magnification) after controlled inflation, fixation and staining with hematoxylin-eosin. C57Bl/6 mice were exposed to ambient air (AC) or chronic cigarette smoke (CS) for 3 months (A-B), 6 months (C-D), or 9 months (E-F), or 6 months followed by 3 months recovery at ambient air (G). Images are representative of n = 5–10 mice /group.
(TIFF)

S3 Fig Metabolomic detection of cigarette exposure in mice. **(A)** Hydroxycotinine in mouse plasma at 6 air control and 6 cigarette smoking time points, and following smoking cessation. **(B)** 4-Oxo-4-(3-pyridyl)-butanoic acid in mouse BAL fluid at two air control and 2 cigarette smoking time points, and following smoking cessation. Samples were analyzed using LC-MS metabolomics. The missing values in some animals are likely due to matrix interference or other limitations in instrument sensitivity; these are occasionally seen in untargeted approaches such as used here. The x-axis represents the smoking status where AC = air control, CS = cigarette smoke exposed, SS = stop smoking, and time point where D = day, M = month.
(TIFF)

S4 Fig Heatmap of GAGE gene set statistics. All pathways in figure were significantly perturbed at more than 1 time point (FDR≤0.10). An asterisk indicates pathway was enriched for upregulated genes after 1 day CS exposure, then enriched for downregulated genes after 7 days CS exposure; † indicates pathway enriched for upregulated genes after 1 day CS exposure, then enriched for downregulated genes after 7 days and later in the time course as well; ‡ indicates pathway enriched for downregulated genes after 1 day CS, then enriched for upregulated genes after 7 days and 9 month of CS exposure.
(TIFF)

S5 Fig DEXSeq transcript view of *Agpat3*. Significant differential usage was found for the exon of *Agpat3* labeled E018 in the figure. The transcript (ENSMUST00000105389) that supports this exon is in the 6th row, and the exon is untranslated (open box). E018 is highlighted in pink.
(TIFF)

S6 Fig Pyrimidine metabolism pathway. The boxes represent genes involved in the KEGG pathway, and the circles represent metabolites, gene products, or chemical compounds. Bright green color indicates that the gene was downregulated in CS-exposed mice, and red indicates the gene was upregulated in CS-exposed mice. Grey indicates very little differential gene expression, and white boxes indicate that expression data for that gene was not available to evaluate the significance of the pathway.
(TIFF)

S7 Fig Glutathione metabolism pathway. The boxes represent genes involved in the KEGG pathway, and the circles represent metabolites, gene products, or chemical compounds. Bright green color indicates that the gene was downregulated in CS-exposed mice, and red indicates the gene was upregulated in CS-exposed mice. Grey indicates very little differential gene expression, and white boxes indicate that expression data for that gene was not available to evaluate the significance of the pathway.
(TIFF)

## Abbreviations

AC: air control
COPD: chronic obstructive pulmonary disease
CS: cigarette smoke
PI: phosphatidylinositol
