# Supplementary material for: Gene and metabolite time-course response to cigarette smoking in mouse lung and plasma
Source: PLoS One. 2017 Jun 2;12(6):e0178281. doi: 10.1371/journal.pone.0178281 (PMC5456044; doi:10.1371/journal.pone.0178281)
Supplement: S1 File — (DOCX) [file pone.0178281.s001.docx]

Online Data Supplement

Global gene and metabolite time course response to cigarette smoke in a mouse model

Mikaela A. Miller^1^

Thomas Danhorn^2^

Charmion I. Cruickshank-Quinn^3^

Sonia M. Leach^2^

Sean Jacobson^5^

Matthew J. Strand^4^

Nichole A. Reisdorph^3^

Russell P. Bowler^5,#^

Irina Petrache^5,6,#^

Katerina J. Kechris^1,#^

1. Department of Biostatistics & Informatics, Colorado School of Public Health, University of Colorado Denver, Aurora, CO
2. Center for Genes, Environment, and Health, National Jewish Health, Denver, CO
3. Department of Pharmaceutical Sciences, School of Pharmacy, University of Colorado Denver, Aurora, CO
4. Division of Biostatistics and Bioinformatics, National Jewish Health, Denver, CO
5. Division of Pulmonary, Critical Care and Sleep Medicine, National Jewish Health and University of Colorado, Denver, CO
6. Department of Medicine, Indiana University, Indianapolis, IN

Running Head: Sustained smoking-induced gene responses

#Address correspondence to Irina Petrache, Russell P. Bowler, or Katerina Kechris

Email: [PetracheI@njhealth.org](mailto:PetracheI@njhealth.org); [BowlerR@njhealth.org](mailto:BowlerR@njhealth.org); [katerina.kechris@ucdenver.edu](mailto:katerina.kechris@ucdenver.edu)

Phone: 303-270-2080; 303-270-2014; 303- 724-4363

**Extended Materials and Methods**

**RNA extraction**

Total RNA was isolated from whole lung with an RNeasy kit (Qiagen, Valencia, CA, USA).

**RNA-seq library preparation and sequencing**

Before gene filtering and removal of samples not passing quality control, the total number of reads per sample ranged from 919,629 to 45,428,692, with a median value of 31,829,769.

**Bioinformatics Analysis**

Sequence quality was assessed with FastQC version 0.10.1, and by counting the number of reads failing to align with the UCSC mm10 reference genome at 5% mismatch tolerance and by quantifying reads mapping uniquely to intergenic and intronic locations (1-3). Reads overlapping rRNA and tRNA genes were quantified with gene coordinates from the repeat-masking track from the UCSC mm10 annotation (2).

Sequences were mapped to mouse mm10 reference genome using GSNAP version 2013-11-27 with SNP data version 137 and splice sites compatible with the refGene annotation, as well as detection of novel splice sites (3-5). Uniquely mapped reads were quantified with HTSeq package version 0.5.4p3 in the “intersection-nonempty” mode (6). HTSeq was used to map reads to exons, exons to transcripts, and transcripts to genes. For DEXSeq, the reads were reassigned to exon bins with HTSeq 0.6.0 and the scripts provided by DEXSeq. The Ensembl version 78 gene IDs were used since this annotation is more comprehensive and able to capture many more alternative splicing events, but at the expense of statistical power to correct for multiple testing (7). A total of 24,272 genes met quality control standards for further analysis.

Two samples were dropped due to low sequencing quality. Sample 121 (AC, 9 months) was dropped due to extremely low read counts and having 17% of counts unmapped. Sample 150 (CS, 1 day) was also dropped due to having more translocations (mapped reads begin on one chromosome and end on another), more multi-mapping, and extremely high rRNA/tRNA content. One additional sample (95, AC, 6 months) was dropped due to possible contamination by human sequences. For quality reasons, all 1 month data were dropped from the analysis. Sample 43 (AC, 1 month) and sample 49 (CS, 1 month) were potential outliers that exhibited moderate multi-mapping and low exonic mapping percentages. Two other samples from the 1-month time point (42, AC and 50, CS) also showed high ribosomal contents and elevated multi-mapping.

**Statistical Analysis**

HTSFilter (version 1.4.0), a data driven method for filtering low expression genes, calculates the Jaccard similarity between all pairs of samples within the same condition at a variety of normalized-count thresholds and averages the indices for both conditions together. The threshold that maximizes the global similarity index is then used as a threshold for filtering (8). The normalization method used was “DESeq” (version 1.16.0), employed from within the HTSFilter function. We used the default parameter settings for choosing thresholds to evaluate (s.min=1, s.max=200, s.length=100).

***Individual time point analysis***

All individual genes were termed “statistically significant” if they exhibited an FDR-adjusted *q*-value less than or equal to 0.10. Because the implicit normalization procedure of edgeR (version 3.10.5) varied slightly with the SS mice included, the lists of differentially expressed genes and significantly perturbed pathways between AC- and CS-exposed mice differed slightly. There were 862 differentially expressed genes at month 9 between CS and AC in the individual time point analysis, and 882 in the cessation analysis. There were 781 genes overlapping between these two lists. There were 20 and 24 pathways enriched for upregulated genes in the individual time points and smoking cessation analysis results, respectively, with an overlap of 20 pathways. There were 35 and 29 pathways enriched for downregulated genes in the individual time points and smoking cessation analysis results, respectively, with an overlap of 29 pathways.

***Functional annotation and pathway analysis***

We used GAGE (version 2.18.0) for gene set enrichment analysis, which uses the fold change ranking of all genes in all pairwise comparisons across experimental groups. GAGE requires normalized read counts, and edgeR does not transform the data in this way but instead uses model specification to correct for transcript abundance. For this reason, raw read counts were normalized using the DESeq2 (version 1.8.2) normalization method and transformed using the Variance Stabilizing Transformation (VST) within DESeq2. This method was chosen over the joint edgeR workflow with GAGE since the one-on-one comparisons are more powerful than using mean fold changes (9). All pathways termed “statistically significant” exhibited an FDR-adjusted *q*-value less than or equal to 0.10. “Influential genes” were determined using the coreGeneSets functionality in GAGE (9).

***Time series analysis***

Gene-wise linear models were fit for gene expression variables using time and group as categorical predictors, including group-by-time interaction in a reference model. A protected testing procedure was used to test first for the overall significance of time and group-by-time interactions (FDR≤0.10). Only those genes passing the protected testing procedure with significant overall effects for time or group-by-time interactions were subjected to the polynomial tests. Orthonormalized polynomial contrasts designed with appropriate coefficients to accommodate unevenly spaced time points were utilized to test linear and quadratic trends for time as well as polynomial interactions between group and time (10). A more stringent cutoff (unadjusted *p*<0.001) was used to test for significant polynomial trends and interactions to further reduce potential false positives and limit the results to the most significant effects.

***Metabolomics analysis***

In contrast to the transcriptome data, which was generated from extracted RNA from lung tissue, the metabolomic data was generated from plasma and broncho-alveolar lavage fluid (BALF) samples. Raw data was generated in triplicates and peak identification and integration was performed using Mass Hunter software (Agilent) as previously described (11). Metabolites were aligned based on mass and retention time, and annotated using IDBrowser within Mass Profiler Professional (MPP). Downstream processing was then performed using the R package MSPrep (12). A maximum coefficient of variation of 0.5 was set as a threshold for utilizing the mean of the triplicates or the median of the triplicates and each compound must have been identified in at least 2 of 3 triplicates in order to summarize the triplicates and obtain one measurement per compound per sample. The data was then filtered to only compounds that were found in at least 80 percent of samples. Missing data was then imputed using Bayesian Principal Components Analysis (BPCA), a Bayesian estimation procedure that is not sensitive to the level of missing data and which uses linear combinations of principal axis vectors to estimate the missing values (13). Student’s t-tests (unadjusted *p*≤0.05) were performed to determine statistically significant metabolites between sample groups. These metabolites were then imported into KEGG Mapper to identify perturbed metabolic pathways. Analysis specific to the BALF metabolome is described below.

***Metabolomics Analysis of Bronchial Alveolar Lavage Fluid (BALF)***

Metabolomics data was obtained using the same methods as plasma. Then, metabolites were filtered for presence in at least 50% of a group. Statistical analysis was performed in Mass Profiler Professional (MPP, Agilent Technologies) using either T tests or ANOVA with Tukey HSD to compare groups. Multiple testing correction was performed using Storey with Bootstrapping q-value < 0.05, and the statistically significant and annotated metabolites were mapped to biological pathways. Pathway enrichment was performed using metabolites biological role (MBROLE) (14)

***Mouse-Human Comparison for Smoking Cessation***

We searched NCBI/GEO for human lung gene expression studies comparing former with current smokers. Only one dataset had at least 10 samples for each group in lung (GSE994). For this dataset, we tested orthologous human genes for differences between former smokers and current smokers and compared those with the mouse genes that showed differences between the smoking cessation (SS) and the cigarette smoke (CS) model. We used Biomart Ensembl <http://uswest.ensembl.org/biomart/martview/> to map human to mouse genes. Then, we calculated p-values in the human dataset for comparison with mouse p-values using an independent 2-group t-test in the statistical software R between former smokers and current smokers for gene expression values and compared those to the SS vs CS results in mouse. When multiple probesets in the downloaded human data mapped to the same gene symbol, we used the probeset with the smallest p-value. We then matched our human genes to mouse genes using the Ensembl ortholog mapping tool. When multiple mouse genes mapped to the same human gene in Ensembl, we used the minimum p-value across the mouse genes. Unlike the other gene lists presented for mouse, these comparisons are not restricted to genes with fold change > 1.5 to not limit comparisons between human and mouse. However, genes are only kept if they are in the same direction (higher/lower in SS vs CS compared to higher/lower in former vs current smokers), and we use a p-value cutoff of 0.022 for both human and mouse (which is equivalent to the FDR cutoff of 0.10 from Table E9 for the SS vs CS comparison).

***Mouse-Human Comparison for Emphysema***

We identified four studies through a NCBI/GEO query search for human, emphysema and lung expression (GDS670 Golpon et al 2004 (15); GDS737 Spira et al. 2004 (16); GSE17770 Francis et al. 2009 (17), GSE27597 Campbell et al. 2012 (18)). Articles returned from the search that related to other lung diseases were removed from our query. We took genes symbols provided in the supplemental tables of these papers, comparing emphysema to controls, and found those genes that overlapped with the orthologous gene symbol in mouse using the method described above. Overlapping gene symbols between human and mouse were reported regardless of the direction of the fold change in the human study, which was not always provided in the supplemental tables from the original references. Supplemental Table E11 includes relevant columns from the supplemental tables from the human study references, in addition to a summary of the mouse results from this work.

Bibliography

1. Bioinformatics B. FastQC: A quality control tool for high throughput sequence data 2015. Available from: <http://www.bioinformatics.babraham.ac.uk/projects/fastqc/>.

2. Quinlan AR, Hall IM. BEDTools: a flexible suite of utilities for comparing genomic features. Bioinformatics. 2010;26(6):841-2. doi: 10.1093/bioinformatics/btq033. PubMed PMID: 20110278; PMCID: PMC2832824.

3. Rosenbloom KR, Armstrong J, Barber GP, Casper J, Clawson H, Diekhans M, Dreszer TR, Fujita PA, Guruvadoo L, Haeussler M, Harte RA, Heitner S, Hickey G, Hinrichs AS, Hubley R, Karolchik D, Learned K, Lee BT, Li CH, Miga KH, Nguyen N, Paten B, Raney BJ, Smit AF, Speir ML, Zweig AS, Haussler D, Kuhn RM, Kent WJ. The UCSC Genome Browser database: 2015 update. Nucleic Acids Res. 2015;43(Database issue):D670-81. doi: 10.1093/nar/gku1177. PubMed PMID: 25428374; PMCID: PMC4383971.

4. Wu TD, Nacu S. Fast and SNP-tolerant detection of complex variants and splicing in short reads. Bioinformatics. 2010;26(7):873-81. doi: 10.1093/bioinformatics/btq057. PubMed PMID: 20147302; PMCID: PMC2844994.

5. Wu TD, Watanabe CK. GMAP: a genomic mapping and alignment program for mRNA and EST sequences. Bioinformatics. 2005;21(9):1859-75. doi: 10.1093/bioinformatics/bti310. PubMed PMID: 15728110.

6. Anders S, Pyl PT, Huber W. HTSeq--a Python framework to work with high-throughput sequencing data. Bioinformatics. 2015;31(2):166-9. doi: 10.1093/bioinformatics/btu638. PubMed PMID: 25260700; PMCID: PMC4287950.

7. Hooper JE. A survey of software for genome-wide discovery of differential splicing in RNA-Seq data. Hum Genomics. 2014;8:3. doi: 10.1186/1479-7364-8-3. PubMed PMID: 24447644; PMCID: PMC3903050.

8. Rau A, Gallopin M, Celeux G, Jaffrezic F. Data-based filtering for replicated high-throughput transcriptome sequencing experiments. Bioinformatics. 2013;29(17):2146-52. doi: 10.1093/bioinformatics/btt350. PubMed PMID: 23821648; PMCID: PMC3740625.

9. Luo W, Friedman MS, Shedden K, Hankenson KD, Woolf PJ. GAGE: generally applicable gene set enrichment for pathway analysis. BMC Bioinformatics. 2009;10:161. doi: 10.1186/1471-2105-10-161. PubMed PMID: 19473525; PMCID: PMC2696452.

10. Hedeker DR, Gibbons RD. Longitudinal data analysis. Hoboken, N.J.: Wiley-Interscience; 2006. xx, 337 p. p.

11. Cruickshank-Quinn CI, Mahaffey S, Justice MJ, Hughes G, Armstrong M, Bowler RP, Reisdorph R, Petrache I, Reisdorph N. Transient and persistent metabolomic changes in plasma following chronic cigarette smoke exposure in a mouse model. PLoS One. 2014;9(7):e101855. doi: 10.1371/journal.pone.0101855. PubMed PMID: 25007263; PMCID: PMC4090193.

12. Hughes G, Cruickshank-Quinn C, Reisdorph R, Lutz S, Petrache I, Reisdorph N, Bowler R, Kechris K. MSPrep--summarization, normalization and diagnostics for processing of mass spectrometry-based metabolomic data. Bioinformatics. 2014;30(1):133-4. doi: 10.1093/bioinformatics/btt589. PubMed PMID: 24174567; PMCID: PMC3866554.

13. Oba S, Sato MA, Takemasa I, Monden M, Matsubara K, Ishii S. A Bayesian missing value estimation method for gene expression profile data. Bioinformatics. 2003;19(16):2088-96. PubMed PMID: 14594714.

14. Chagoyen M, Pazos F. MBRole: enrichment analysis of metabolomic data. Bioinformatics. 2011;27(5):730-1. doi: 10.1093/bioinformatics/btr001. PubMed PMID: 21208985.

15. Golpon HA, Coldren CD, Zamora MR, Cosgrove GP, Moore MD, Tuder RM, Geraci MW, Voelkel NF. Emphysema lung tissue gene expression profiling. Am J Respir Cell Mol Biol. 2004;31(6):595-600. doi: 10.1165/rcmb.2004-0008OC. PubMed PMID: 15284076.

16. Spira A, Beane J, Pinto-Plata V, Kadar A, Liu G, Shah V, Celli B, Brody JS. Gene expression profiling of human lung tissue from smokers with severe emphysema. Am J Respir Cell Mol Biol. 2004;31(6):601-10. doi: 10.1165/rcmb.2004-0273OC. PubMed PMID: 15374838.

17. Francis SM, Larsen JE, Pavey SJ, Bowman RV, Hayward NK, Fong KM, Yang IA. Expression profiling identifies genes involved in emphysema severity. Respir Res. 2009;10:81. doi: 10.1186/1465-9921-10-81. PubMed PMID: 19723343; PMCID: PMC2746189.

18. Campbell JD, McDonough JE, Zeskind JE, Hackett TL, Pechkovsky DV, Brandsma CA, Suzuki M, Gosselink JV, Liu G, Alekseyev YO, Xiao J, Zhang X, Hayashi S, Cooper JD, Timens W, Postma DS, Knight DA, Lenburg ME, Hogg JC, Spira A. A gene expression signature of emphysema-related lung destruction and its reversal by the tripeptide GHK. Genome Med. 2012;4(8):67. doi: 10.1186/gm367. PubMed PMID: 22937864; PMCID: PMC4064320.
