# Supplementary figures and images for: Gene and metabolite time-course response to cigarette smoking in mouse lung and plasma
Source: PLoS One. 2017 Jun 2;12(6):e0178281. doi: 10.1371/journal.pone.0178281 (PMC5456044; doi:10.1371/journal.pone.0178281)

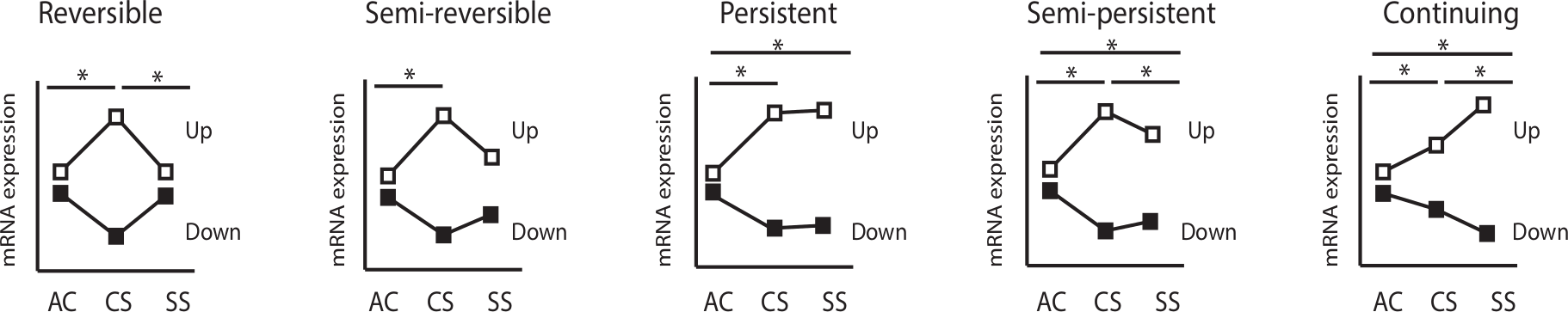

Supplement: S1 Fig — Within each pattern, the white squares represent a pattern of upregulation in CS- vs. AC-exposed mice, and black squares represent a pattern of downregulation in CS- vs. AC-exposed mice. Comparisons with asterisks represent differences that must be statistically significant for the cessation pattern to hold. We defined as fully reversible genes those that were significantly differentially expressed between in CS-exposed mice compared to AC and that were differentially expressed in SS (stop smoking or smoking cessation) in an opposite direction compared to CS-exposed mice, but not differentially expressed in SS compared to AC. Compared to fully reversible patterns, semi-reversible genes did not fully return to AC levels, being differentially expressed between AC and SS mice. Fully persistent genes were significantly differentially expressed in CS vs. AC, and AC vs. SS, but not between SS and CS mice. Semi-persistent genes were similar to fully reversible genes, except they were differentially expressed between CS and SS mice. Continuing patterns were defined as higher differential expression in AC vs. SS comparison compared to the AC vs. CS comparison. (TIFF) [file pone.0178281.s003.tiff]

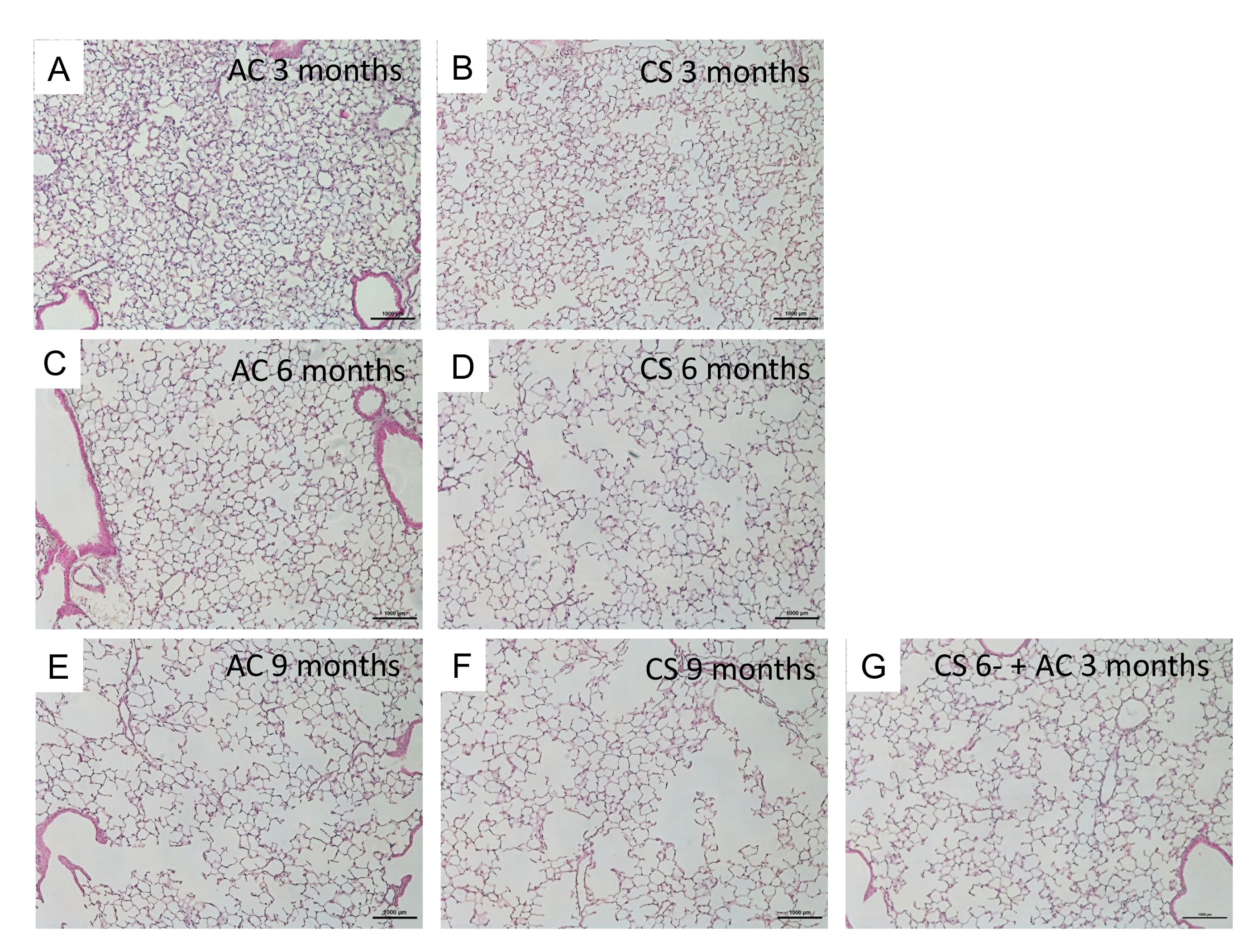

Supplement: S2 Fig — Airspaces of mouse lungs imaged (10x magnification) after controlled inflation, fixation and staining with hematoxylin-eosin. C57Bl/6 mice were exposed to ambient air (AC) or chronic cigarette smoke (CS) for 3 months (A-B), 6 months (C-D), or 9 months (E-F), or 6 months followed by 3 months recovery at ambient air (G). Images are representative of n = 5–10 mice /group. (TIFF) [file pone.0178281.s004.tiff]

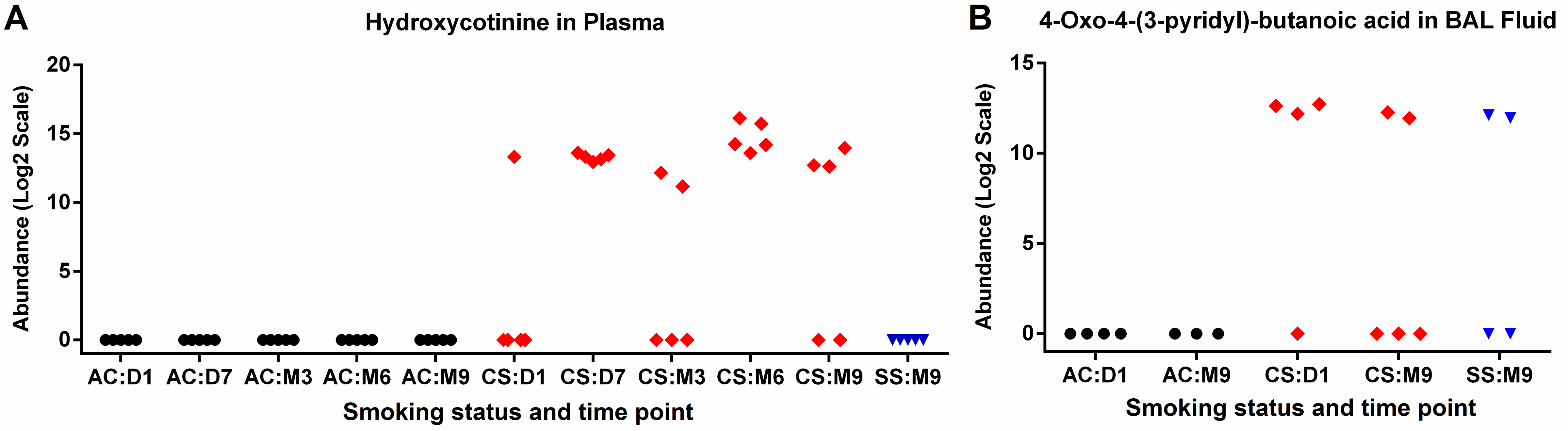

Supplement: S3 Fig — (A) Hydroxycotinine in mouse plasma at 6 air control and 6 cigarette smoking time points, and following smoking cessation. (B) 4-Oxo-4-(3-pyridyl)-butanoic acid in mouse BAL fluid at two air control and 2 cigarette smoking time points, and following smoking cessation. Samples were analyzed using LC-MS metabolomics. The missing values in some animals are likely due to matrix interference or other limitations in instrument sensitivity; these are occasionally seen in untargeted approaches such as used here. The x-axis represents the smoking status where AC = air control, CS = cigarette smoke exposed, SS = stop smoking, and time point where D = day, M = month. (TIFF) [file pone.0178281.s005.tiff]

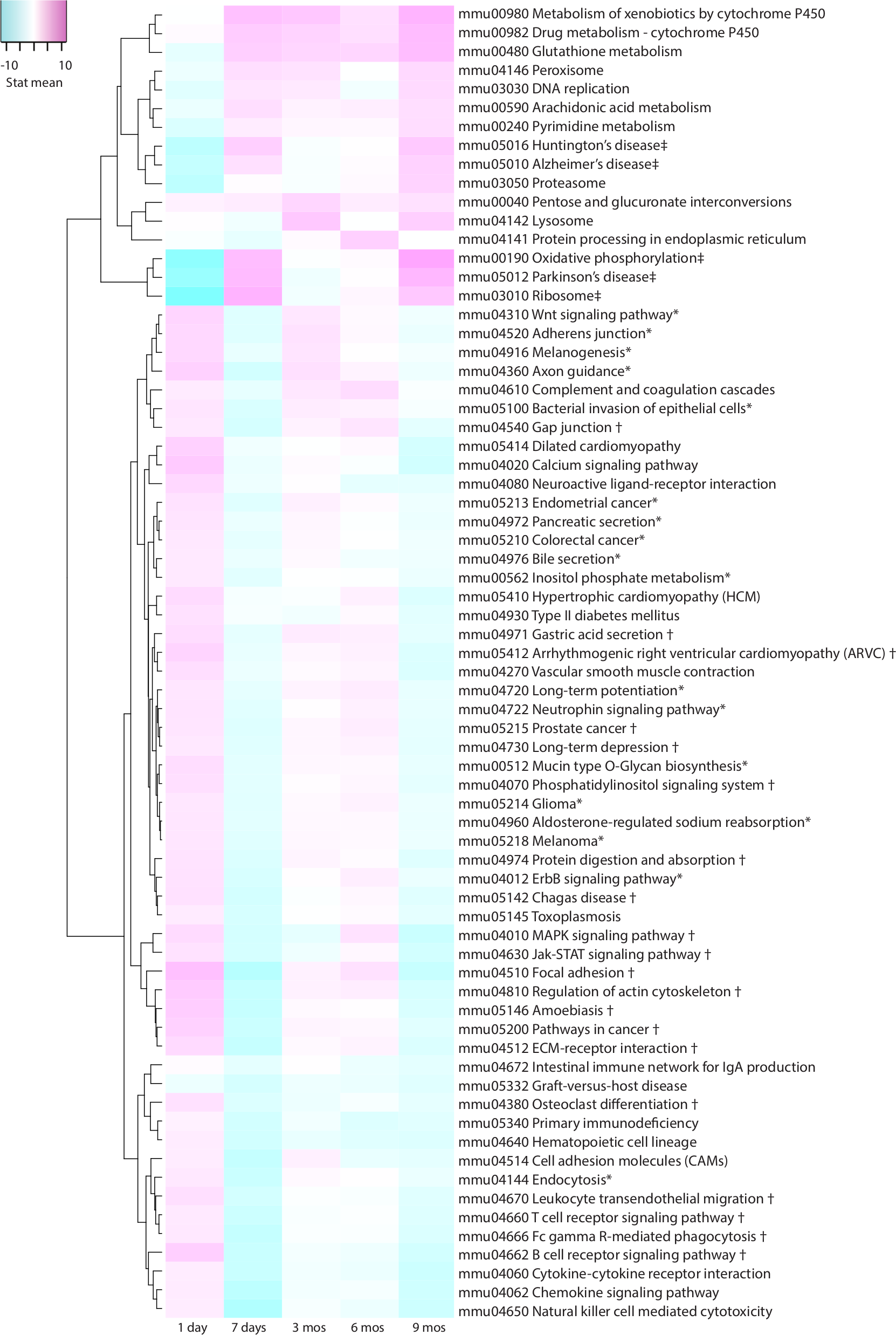

Supplement: S4 Fig — All pathways in figure were significantly perturbed at more than 1 time point (FDR≤0.10). An asterisk indicates pathway was enriched for upregulated genes after 1 day CS exposure, then enriched for downregulated genes after 7 days CS exposure; † indicates pathway enriched for upregulated genes after 1 day CS exposure, then enriched for downregulated genes after 7 days and later in the time course as well; ‡ indicates pathway enriched for downregulated genes after 1 day CS, then enriched for upregulated genes after 7 days and 9 month of CS exposure. (TIFF) [file pone.0178281.s006.tiff]

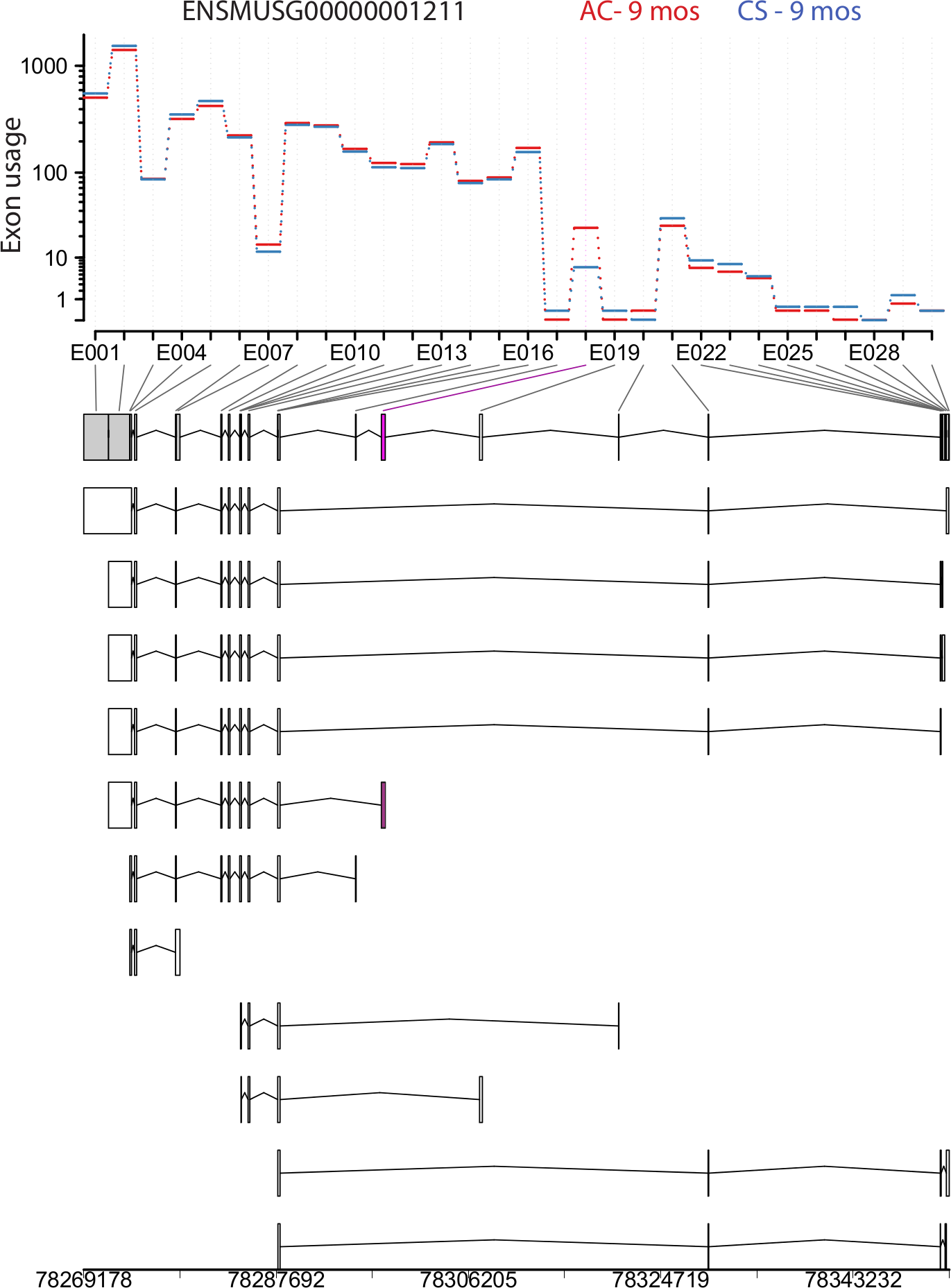

Supplement: S5 Fig — Significant differential usage was found for the exon of Agpat3 labeled E018 in the figure. The transcript (ENSMUST00000105389) that supports this exon is in the 6th row, and the exon is untranslated (open box). E018 is highlighted in pink. (TIFF) [file pone.0178281.s007.tiff]

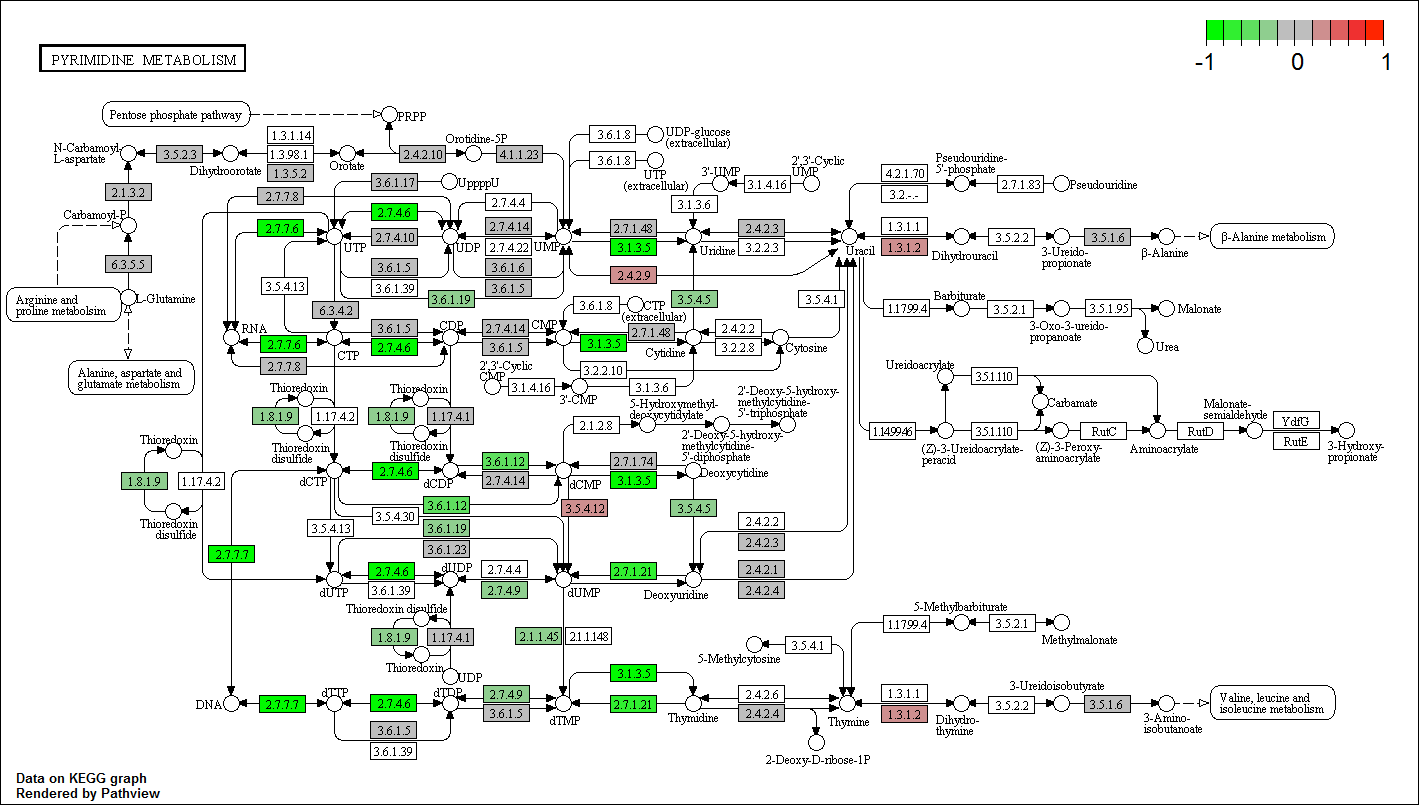

Supplement: S6 Fig — The boxes represent genes involved in the KEGG pathway, and the circles represent metabolites, gene products, or chemical compounds. Bright green color indicates that the gene was downregulated in CS-exposed mice, and red indicates the gene was upregulated in CS-exposed mice. Grey indicates very little differential gene expression, and white boxes indicate that expression data for that gene was not available to evaluate the significance of the pathway. (TIFF) [file pone.0178281.s008.tiff]

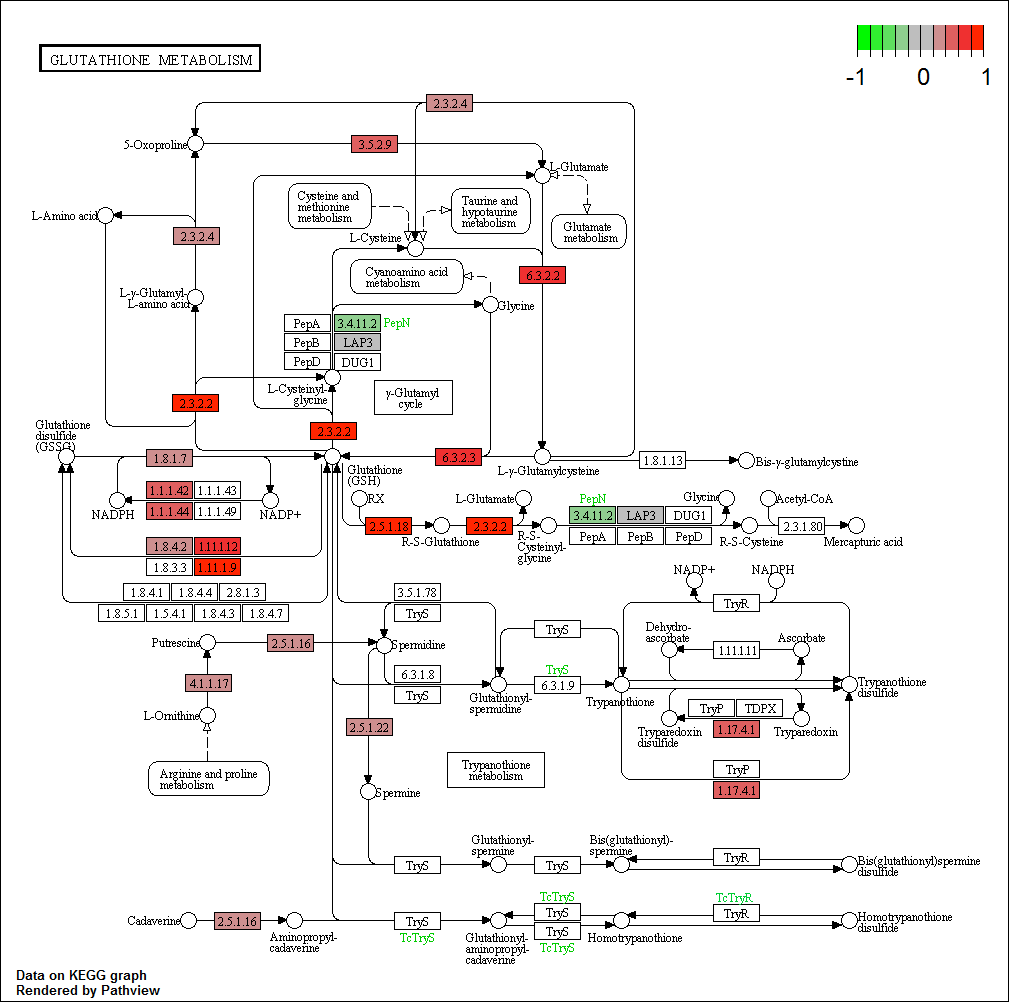

Supplement: S7 Fig — The boxes represent genes involved in the KEGG pathway, and the circles represent metabolites, gene products, or chemical compounds. Bright green color indicates that the gene was downregulated in CS-exposed mice, and red indicates the gene was upregulated in CS-exposed mice. Grey indicates very little differential gene expression, and white boxes indicate that expression data for that gene was not available to evaluate the significance of the pathway. (TIFF) [file pone.0178281.s009.tiff]
